# International Dental Federation and International Commission of Education: Meeting Held in Stockholm, Sweden, August, 1902

**Published:** 1903-06

**Authors:** 


### INTERNATIONAL DENTAL FEDERATION AND INTERNATIONAL COMMISSION OF EDUCATION: MEETING HELD IN STOCKHOLM, SWEDEN, AUGUST, 1902.

(Continued from page 388.)

OK

### REPORTS ON DENTAL EDUCATION (CONTINUED).

In addition to the reports on dental education presented by various national representatives on Monday, August 18, as already published (see page 371), the Commission of Education received those here given.

The following is a summary of the report presented by Edmund Rosenthal, D.M.D., on behalf of Belgium:

In answer to the first question, which reads, "What are the preliminary studies to be required of students before beginning their professional education?" Dr. Rosenthal said that in his belief it is desirable that students should receive a complete preliminary education of a high standard, and, above all, a useful one. He recommended that the education of the candidate should be carried on in such a way as to insure perfect intellectual development. He should know the literature of different periods and should be conscious of the progressive changes which the human race has been undergoing in order to reach the relatively perfect stage of civilization in which it finds itself at present. He advocated the study of living languages so that the student may readily become acquainted with the nature of the work that may be doing and the progress achieved in the different countries of the globe. He wisely called attention to the fact that the antagonism that has been exhibited towards the dental profession has been generally directed to the lack of proper literary and scientific training of dentists, while their technical ability has never been questioned.

He did not deem it necessary to enter into a discussion of the studies that should be included in the preliminary curriculum, not because he approves of the educational programmes in force at present in different countries, but because by following them the dental candidate is put upon the same footing as the students of the other liberal professions. But besides these studies the future dentist should be made to develop his manual ability from an early age. Manual ability can be acquired. It is not indispensable that dexterity should be an innate quality, for even those that are not naturally so inclined can in time become reasonably skilful. He attaches a great deal of importance to the study of drawing, especially drawings of the head from nature. Every dentist should be able to draw the ideal facial lines of a given type according to its origin, temperament, and special characteristics. The sooner the manual and artistic education is undertaken the more profitable it will be, for by following this plan it becomes possible to devote a great deal more time to the theoretical branches without detriment to the technical ones. The young man who at the age of twenty-two or twenty-three is not capable of using his fingers for anything beyond holding a pen is certainly in need of a professional apprenticeship of longer duration than is the one who has applied himself to the development of his manual and artistic faculties, and who is accustomed to observe and compare, and whose fingers are trained to carry out the suggestions of his brain. It matters not in what direction this manual ability is trained; so long as it exists it can be directed at any time to any line of work; but, if it be wanting, a considerable period of time is necessary to acquire it, and the practitioner will always regret having undertaken too late the manual education necessary to perform the work pertaining to his profession. In carrying out the duties of his calling the dentist is called upon to play the part of artist, sculptor, and creator, inasmuch as he has to modify disfigured faces and to repair the ravages of age. If guided by routine only,—that is, by a mere utilitarian comprehension of his profession,—he certainly may render useful service, but he will neglect the æsthetic side and will never experience the high satisfaction of executing perfect work from the point of view embracing both the artistic and the useful.

The practitioner who applies himself to the study of nature and strives to imitate her best work ceases to be the handicraftsman and becomes the artist in the full sense of the term; but, with rare exceptions, one cannot become either an artist or a conscientious practitioner without developing the germs of art which exist in every man. Through the manipulation of clay by moulding heads after nature and according to selected models that impress upon the mind the lines of the human face, and by classifying these observations, it becomes possible to determine the expression to be given to the edentulous face in order to restore the original beauty that is now absent.

Discussing the second question, which reads, "In what are dental studies to consist, how long ought they to last, and what should be the order of the programme?" he said that the course should be one of four years. The subjects of the first year should be: Chemistry, physics, botany, mineralogy, metallurgy, a considerable number of practical exercises, dental prosthesis, drawing, and modelling. Second year: General anatomy, physiology, embryology, pharmacodynamics, general histology, practical work in microscopy, dental prosthesis. Third year: Special comparative anatomy of the mouth, general and special pathology, pathological anatomy, surgical and medical pathology, operative dentistry, practical work in operative dentistry, pathology, microscopy, and dental prosthesis. Fourth year: Medical and surgical clinics, theory and practice of surgical operations, orthodontia, operative dentistry, general hygiene in its relation to dentistry, oral hygiene, physiological and pathological analysis of human secretions, saliva, urine, etc., practical work in operative dentistry, prosthetic dentistry, facial restorations, bridge-work, and porcelain work.

In answer to the third question, " What branches of the studies taught in medical schools must the student of dentistry follow?" the essayist said that, with the exception of the practical work in the medical and surgical clinics, all the branches of the four years' curriculum should be given in the dental school according to special programmes, but as dental schools are not often in the position tc afford the material equipment necessary for the complete education of the dentist, the dental student will have to pursue in the medical schools those courses in science for which the medical laboratories are especially adapted, possessing as they do the appliances and collections of specimens suited to and indispensable for this kind of work. From the fact that part of the staff of the universities and medical schools devote their time exclusively to the study of science and to laboratory work, they are better fitted, in the essayist's estimation, for imparting such instruction than men who have divided their time between teaching and practising.

Professor Dr. Hesse, of Leipzig, presented a report on the status of dental education and legislation in Germany, an abstract of which here follows:

In Germany the practice of the healing art is free, and the only legal restrictions apply to the use of the titles of *Arzt* and *Zahnarzt*, which belong only to the candidates who have successfully passed the examinations leading to those degrees. Referring to the dental institutions of Germany, he considered it necessary to say a few words regarding their organization, as the several communications which have been lately published by Dr. W. C. Barrett show that foreigners are lacking completely in information on this question. In Germany all professors of dentistry are members of university staffs and are part of the faculties of medicine. Their special work consists in the theoretical and practical teaching of dentistry, and they are in this particular sense absolutely independent. The study of natural and medical sciences is carried out in the university under the direction of the faculty of medicine. Apparently it is this arrangement which has caused Dr. Barrett to believe that our education in the practical branches is inferior to that of the United States. We believe, however, that our organization offers a great advantage, as it permits of a thorough specialization and concentration of the teaching staff to dental science and art; at the same time our students have the opportunity of becoming familiarized with medicine,—an association which we consider important enough to recommend its consideration in the countries in which education is not carried out on this plan.

He then refers to the conditions of admission to examinations leading to the degree of *Zahnarzt,* and describes in detail the various topics upon which the student is examined.

Dr. Guillermin presented the following report:

REPORT ON THE THREE QUESTIONS PROPOSED BY THE INTERNATIONAL COMMISSION OF EDUCATION OF THE INTERNATIONAL DENTAL FEDERATION.

*Question 1.*—"What are the preliminary studies to be required of students before beginning their professional education?"

Two kinds of studies must be considered,—namely, (*a*) the secondary education, and (*b*) the university theoretical or superior studies.

(*a*) The secondary (preliminary) studies should be the same as for the study of medicine.

(*b*) The superior studies, in my estimation, should embrace the complete study of medicine, but it would be desirable in view of its extensive scope to limit the course in medicine to four years. This would constitute not the basis of the cone as advocated by Sir Michael Foster in Cambridge, but the trunk from which would branch out the different specialties, otology, laryngology, ophthalmology, stomatology, etc.

If the organization of dental education upon this basis could not be carried out, the plan adopted for the obtaining of the Swiss federal diploma is the one that we would advocate. In this curriculum one year is devoted to natural sciences (physics, inorganic and organic chemistry, botany zoölogy, and comparative anatomy), one to the medical sciences (anatomy with dissection of the muscles, vessels, and nerves of the head and neck; practical work in microscopy and theoretical anatomy), histology, embryology, and physiology in their bearing on dentistry; practical courses in general pathology and pathological anatomy and in special pathology and therapeutics of the mouth; attendance during two semesters upon one of the surgical clinics.

*Question 2.*—"Of what are dental studies to consist, how long ought they to last, and what should be the order of the programme?"

After the pursuance of the theoretical studies above indicated, a minimum of two semesters should be devoted to clinical dentistry, three semesters to prosthetic dentistry, and three semesters to operative dentistry. In Switzerland the law regulating medical examinations allows the three semesters on prosthetic dentistry and the three semesters on operative dentistry to be taken in the laboratory and in the office of a licensed dentist.

In the dental school of Geneva the curriculum is so arranged that the professional studies may not take up more than three semesters. In some countries, as well as in some of the cantons of Switzerland, the diploma of physicians gives *ipso facto* the right to practise dentistry. This is a provision which we disapprove of, as we believe that physicians should not be permitted to enter upon the practice of dentistry unless they have pursued three semesters of its professional studies.

This report was followed by one on "Dental Legislation in Switzerland," by the same author. A *résumé* of this report here follows:

In the canton of Geneva the dental school grants the diploma of *médecin-chirurgien-dentiste,* which has now been substituted by the licentiate in dental surgery. This diploma confers by itself the right to practise dentistry, but only in the canton of Geneva. Incidentally it may be stated that the diploma of doctor of medicine of the faculty of Geneva likewise confers only the right to practise medicine in the canton in which it is granted. These diplomas are easier to obtain than the federal diplomas. The dental school of Zurich does not issue diplomas; it merely prepares the students for the federal examinations. The federal diploma is one of the requirements for admission as a regular member of the Swiss Odontological Society. By a singular anomaly in a country as enlightened as Switzerland, one canton, that of Glarus, and two demi-cantons, those of Basle-Campagne and Appenzell, have declared that the practice of medicine and dentistry is free. It must be added that sometimes the authorities of some of the cantons confer the right to practise to holders of foreign diplomas provided that they confer the right to practise in the countries in which they are granted.

Physicians, except in some cantons, as in Zurich and Berne, have likewise the right of practising dentistry without the necessity of any special professional training. Dental education is given in Switzerland in two schools, that of Geneva and that of Zurich. Recently the chair of odontology has been created in the University of Basle. This course comprises (1) pathology and therapeutics of the mouth and teeth; (2) anatomy of the mouth and teeth; (3) diseases of the mouth and teeth; (4) operative dentistry and prosthesis; (5) stomatology. Courses 1, 2, and 5 are given at the university; courses 3 and 4 in the office of the professor.

The following additional report on dental education in Switzerland was presented by the delegates of the Swiss Odontological Society, L. C. Bryan, D.D.S., Theodore Frick, D.D.S., and Paul Guye, D.D.S.:

REPORT ON THE THREE QUESTIONS PROPOSED BY THE INTERNATIONAL COMMISSION OF EDUCATION OF THE INTERNATIONAL DENTAL FEDERATION.

*Question 1.*—"What are the preliminary studies to be required of students before beginning their professional education?"

The preliminary studies should be equivalent to those required from medical students. Considering, however, the importance of languages, the preference should be given to a baccalaureate degree comprising one living language besides the native language of the candidate. It would also be advisable that part of the time should be devoted to drawing and to mathematics.

*Question 2.*—"In what are dental studies to consist, how long ought they to last, and what should be the order of the programme?"

The studies should cover four years. The practical exercises in the laboratories, clinical and otherwise, should take up a large portion of the student's time, and should begin (especially prosthetic dentistry), with the first semester of the course.

The three delegates approved the conclusions presented by Dr. E. D. Rosenthal, D.M.D., Brussels, but stated that besides the topics mentioned by the Belgian delegate, technical requirements as practised to-day in the best schools of America should be included in the programme.

*Question 3.*—" What branches of the studies taught in the medical schools must the student of dentistry follow?"

In order to abbreviate their report, the delegates stated that they fully agreed with the answer to this question as presented by Dr. Rosenthal.

Dr. E. D. Rosenthal, Brussels, Belgium, then presented an additional report, on " The Conditions of Affiliation of all Dental Schools with the International Commission of Education."

Reports on Dental Legislation were presented by Dr. Maurice Roy, France; Dr. Jos. Arkövy, Hungary; Dr. Chas. E. Pearson, Canada; Dr. Weiser, Austria; Dr. E. D. Rosenthal, Belgium; Dr. V. Haderup, Denmark; Professor Limberg, Russia; Drs. L. C. Bryan, Paul Guye, and Theodore Frick, Switzerland.

MONDAY, AUGUST 18.

The Commission was called to order in the Grand Hotel at 9.15 A.M. by the president, Dr. Truman W. Brophy.

The first question proposed by the Federation was brought up for discussion.

Dr. Aguilar proposed the following amendment:

> " In order to be admitted into a dental school, the applicant should have the same preliminary education as required from students of medicine, law, etc., before they are admitted into a university."

Dr. Aguilar was opposed to the suggestion that the student should take a course in manual training before entering the dental school.

Dr. Guerini spoke on the imperative necessity of preserving the high standard of preliminary education.

Dr. Jenkins approved the amendment as presented by Dr. Aguilar.

Dr. Godon stated that a preliminary manual education is necessary, and he opposed Dr. Aguilar's amendment.

Dr. Guilford agreed with Dr. Godon's views concerning the advantages of preliminary manual education for the dentist.

Drs. Guldberg and Förberg were of the opinion that manual training should commence after the entrance of the student into the dental school.

In order to make his amendment applicable to all countries, Dr. Aguilar proposed to add the words "or equivalent" after the word "same."

Dr. Smith considered that this addition was not fully satisfactory.

Dr. Queudot stated that the preliminary education in the case of the dentist should be the same as for other professions.

Dr. Frank was not in favor of Dr. Aguilar's amendment, and asked how this preliminary manual education should be carried out.

On motion of Dr. Harlan, it was decided to refer the amendment presented by Dr. Aguilar to a committee composed of Messrs. Harlan, Godon, Aguilar, Hesse, Harding, and Smith, in order to frame it so that its application to every country should be possible.

President Brophy objected to this amendment, as it would lower the status of dental education in the United States by reason of the inferior standard of some medical schools in that country.

After a long and agitated discussion the question was referred to the next session of the Commission, and the meeting then adjourned.

AFTERNOON SESSION.

The meeting was called to order at three P.M. by President Brophy.

On motion of Dr. Smith, the following answer to the first question was adopted:

> "The same qualifications as are required from students of medicine and of law in those countries in which professional schools are under the supervision of the government, or equivalent qualifications in countries in which such supervision does not exist. The equivalence of these qualifications should be determined by the secretaries of public education of the respective governments."

On motion of Dr. Godon, the question of preliminary manual education was referred for discussion to the next session of the Commission in Madrid.

The Executive Council was directed to prepare the questions to be discussed at the next session of the International Commission of Education.

The report by the secretary-general of the Commission of Education, Dr. Roy, was adopted, and the Executive Council was requested to prepare a report on the same subject to be presented at the next meeting.

The following officers were then unanimously re-elected: Dr. Truman W. Brophy, president; Drs. Patterson, Zsigmondy, Kirk, and Cunningham, vice-presidents; Dr. Roy, secretary; Drs. Frick and Guye, adjunct-secretaries.

President Brophy thanked his colleagues for the support, kindness, and confidence which had been bestowed upon him, and for his re-election as chairman for the ensuing year,—an office which, he said, he hesitatingly accepted. He also thanked his colleagues for their interest and activity in everything concerning the Federation work, and congratulated them upon the results obtained, which, notwithstanding the existence of some differences of opinion because of the differences in the character and nature of dental legislation in different countries, was excellent, promising a great deal for the future.

The meeting then adjourned to meet in Madrid in April, 1903.

